# Quantitative Analysis of SARS-CoV-2 Antibody Status between Patients with Cancer and Healthy Individuals with Extended Vaccination Dosing Intervals in Canada

**DOI:** 10.3390/curroncol29010006

**Published:** 2021-12-24

**Authors:** Andrew Robinson, Andrew Mazurek, Minqi Xu, Yanping Gong

**Affiliations:** 1 Department of Oncology, Kingston Health Sciences Centre, Queen’s University, Kingston, ON K7L 5P9, Canada; Andrew.Robinson@kingstonhsc.ca; 2 Department of Pathology and Molecular Medicine, Kingston Health Sciences Centre, Queen’s University, Kingston, ON K7L 3N6, Canada; 13am175@queensu.ca (A.M.); 18mx5@queensu.ca (M.X.)

**Keywords:** SARS-CoV-2, vaccine, oncology, cancer patients

## Abstract

(1) Background: To date, data addressing the antibody response of cancer patients to SARS-CoV-2 vaccines are limited. To our knowledge, this is the first report to evaluate humoral immunity. responses in Canadian cancer patients. (2) Methods: 116 cancer patients and 35 healthy participants were enrolled in this cross-sectional study. The interval between the first and second doses were closely matched during analysis. IgG antibodies against the SARS-CoV-2 spike receptor–binding domain were determined using an enzyme-linked immunosorbent assay (ELISA). (3) Results: Following two doses of SARS-CoV-2 vaccine (including BNT162b2, AZD1222, and mRNA-1273), the mean serum anti-spike protein antibody level was 382.4 BAU/mL (binding antibody unit, SD ± 9.4) in the control group, 265.8 BAU/mL (±145.7) in solid cancer patients, and 168.2 BAU/mL (±172.9) in hematological cancer patients. Observed differences were significantly lower in both solid and hematological groups when comparing to the control group (*p* ≤ 0.0001). In solid cancer group, patients with cytotoxic chemotherapy demonstrated significantly lower antibody levels (*p* < 0.01), whereas the rest of the patients showed similar antibody levels as the healthy control. Antibody levels were lower in those on treatment than those off treatment in patients with hematological malignancies (*p* < 0.0001) but not for those with solid cancers (*p* = 0.4553). (4) Conclusions: After two doses of the SARS-CoV-2 vaccination, patients with solid and hematological malignancies demonstrated impaired serological responses. This was particularly prominent if there was cytotoxic chemotherapy or systemic therapy in solid and hematological cancer, respectively.

## 1. Introduction

Post COVID-19 immunization, vaccine breakthrough infections occur in a small fraction of all vaccinated persons and account for a small percentage of all COVID-19 cases [1,2,3]. Many factors likely contribute to COVID-19 vaccine breakthrough, such as virus evolution, increased dose of exposure, and poorly developed adaptive immunity in the host. Enzyme-linked immunosorbent assay (ELISA) is the most commonly used methodology to evaluate humoral immunity after immunization. The ELISA based methodology generally outperforms immunochromatographic (ICT) assay for the detection of SARS-CoV-2 antibody due to superior analytical sensitivity and specificity. For most other vaccines, a cut off based on semi-quantitative or quantitative ELISA is often chosen to represent protection and immunity. This cut-off is in the range from 4 to 64 times of seroconversion concentration [4]. The concentration of antibody, which could potentially render protection post-immunization for COVID-19, is not known.

To date, data addressing the antibody response of cancer patients to SARS-CoV-2 vaccines are limited [5,6,7]. In a recent study focused on the Israeli cancer population, 102 adult patients with solid tumors and 78 healthy controls were studied following BNT162b2 vaccination. The median IgG titer in the patients with cancer was significantly lower than that in the controls, following BNT162b2 vaccination during systemic treatment [5]. In a similar study in the UK, measurement of anti-spike protein immunoglobulin levels 21 days following the first dose of the BNT162b2 vaccine showed that 39% of patients with solid cancer, and only 13% of patients with hematological cancer, had developed an adequate level of immune protection, whereas 97% were observed in healthy controls [6]. In Canada, immunization strategies involving vaccine mixing and extended dosing intervals were adopted to increase the availability of vaccines to the general population. This contributed to an added level of complexity when evaluating the effectiveness of protection induced by COVID-19 vaccination in cancer patients [8]. The study objective was to compare humoral immune responses in immunized healthy subjects and immunocompromised cancer patients (either due to treatment regimens or their underlying cancer status), which could provide insights about mechanisms of vaccine breakthrough, at least from a host perspective. To our knowledge, this is the first report to evaluate humoral immune responses in Canadian cancer patients. Our patient population received their second doses on average 50 days after the first dose, which was much longer than the recommended intervals: 21 days for Pfizer-BioNTech and 28 days for Moderna [9,10].

## 2. Materials and Methods

Institutional ethics committee approval and consent from participants were obtained. Participants with known history of COVID-19 infection or with missing data were excluded in the study and analysis. Cancer patients were recruited from the Cancer Centre of Southeastern Ontario and healthy participants were recruited at the Queen’s University using a questionnaire to identify the health status. From May to October 2021 (during the first wave to the beginning of the second wave in Canada), 116 cancer patients and 35 healthy control participants were enrolled in the study. Chart review was performed to determine the types of cancer, treatments, and the timeframe of the treatment.

IgG antibodies against the SARS-CoV-2 spike receptor–binding domain were quantified by ELISA (EUROIMMUN, product number: EI 2606-9601-10). The method has been authorized for Emergency Use Authorization (EUA) by the U.S. Food and Drug Administration. This quantitative method has a linear range between 3.2 to 384 BAU/mL (binding antibody unit). Results below and over the linear ranges were arbitrarily assigned a value of 3.2 and 384 BAU/mL, respectively. A cutoff of 35.2 BAU/mL was used to determine the seroconversion (recommended by the method manufacturer). Two arbitrary cutoffs of 140.8 BAU/mL (4× of seroconversion concentration) and 281.6 BAU/mL (8×) were used to explore the impact of various cutoffs on potential immunity. Statistical analysis was performed using GraphPad Prism version 8.0 (GraphPad, GraphPad Software, San Diego, CA, USA).

## 3. Results

### 3.1. Baseline Characteristics

The baseline characteristics of study participants are summarized in Table 1. All participants received two doses of the vaccine, following the recommended dosing interval in Ontario, Canada. The intervals between the second dose and blood collection were 44 days on average in cancer patients versus 53 days in the healthy control. Due to the limited number of blood samples collected after the first dose, the antibody levels were not compared and discussed.

### 3.2. Treatment Characteristics and Seroconversion Solid Tumour Patients

As demonstrated in Table 2, among patients with solid tumours included in this analysis (*n* = 76), 14 were not actively receiving systemic therapy (off treatments more than 3 months or never received treatment), 30 patients were receiving cytotoxic chemotherapy or cytotoxic chemotherapy plus immunotherapy, 14 were receiving immunotherapy alone, 12 were receiving targeted therapy alone, and 6 were receiving hormonal therapy alone. Many of these patients would have also received corticosteroids as part of their cancer treatment regimen. An examination of the characteristics of steroid use and timing with immunization timing is beyond the scope of this current analysis.

For patients not on therapy (*n* = 14), the median antibody concentration was 381 BAU/mL with a mean of 315 (±156) BAU/mL. Using the manufacturer’s cutoff of 35.2 BAU/mL (as an indication of seroconversion), all 14 patients (100%) seroconverted. Of these patients, 12/14 patients (86%) and 10/14 patients (64%) had levels of 4× and 8× the seroconversion level, respectively. For patients on hormonal therapy alone (*n* = 6), the median antibody concentration was 384 BAU/mL, with a mean of 358 (±64) BAU/mL. All patients seroconverted with a cutoff of 35.2 BAU/mL, and all had levels at least 4× seroconversion concentration. Of these patients, 5/6 patients (83%) had levels 8×. In targeted therapy patients (*n* = 11), the median antibody concentration was 381 BAU/mL, with a mean of 331 (±126) BAU/mL. Seroconversion occurred in 10/11 patients (91%) using the cutoff of 35.2 BAU/mL, while 9 patients (82%) had levels 8× seroconversion. For immunotherapy treated patients without chemotherapy (*n* = 14), the mean antibody concentration was 300 BAU/mL (±138), with a median of 384 BAU/mL. All 14 (100%) seroconverted with the 35.2 BAU/mL cutoff, while 11/14 patients (79%) had levels 4× the conversion level, and 10/14 patients (72%) had levels 8× the conversion level.

For patients on cytotoxic therapy (including cytotoxic therapy plus targeted therapy or cytotoxic therapy plus immunotherapy) (*n* = 30), the mean antibody level was 195 (±156) BAU/mL, with a median antibody level of 154 BAU/mL. Of these patients, 23/30 patients (77%) seroconverted at 35.2 BAU/mL, while 17 patients (57%) had levels 4× the seroconversion level, and 15 patients (50%) had levels 8× the conversion level.

Although the average anti-spike antibody level of all 76 solid cancer patients was significantly lower than that of the healthy control (*p* < 0.0001), only those with cytotoxic chemotherapy showed lower antibody levels (*p* < 0.01), whereas the other four treatment groups showed similar antibody levels. When compared with the patients without active systemic therapy, the other four treatment groups (including cytotoxic chemotherapy) showed similar anti-spike antibody levels (*p* > 0.05).

### 3.3. Treatment Characteristics and Antibody Seroconversion in Hematologic Patients 

As demonstrated in Table 2, for patients with haematologic malignancy (*n* = 40), 13 were receiving no active therapy, 11 were receiving anti-CD20 based therapy (rituximab alone, rituximab-bendamustine, rituximab-CHOP etc), and 16 were receiving systemic therapy but not anti-CD20 therapy.

For patients with hematologic malignancies not receiving any active systemic therapy within 3 months (*n* = 13), the mean antibody level was 340 BAU/mL (±116), with a median of 384 BAU/mL. The seroconversion rate was 93% using the 35.2 BAU/mL cutoff (12/13). Of these patients, 12/13 patients (93%) and 11/13 (86%) had levels of 4× and 8× the seroconversion concentration.

For patients receiving anti-CD20 based therapy (*n* = 11), the median antibody level was 4 BAU/mL, and mean level was 41 (±113) BAU/mL. At the cutoff of 35.2 BAU/mL, only 1 patient of 11 (9%) seroconverted.

For patients with hematologic malignancies receiving systemic therapy excluding anti-CD20 (*n* = 16), the median antibody level was 48 BAU/mL, with a mean of 117 BAU/mL (±144). Eight patients (50%) seroconverted at a minimum 35.2 BAU/mL, while 4 patients (25%) obtained a level of 4× seroconversion, and 3 patients (19%) achieved a level of 8× seroconversion.

The average anti-spike antibody level of all 40 hematologic cancer patients was significantly lower than the healthy control (*p* < 0.0001). When compared to patients without active treatment, patients undergoing both CD-20 and other systemic therapies had lower serological responses (*p* < 0.01).

### 3.4. Overall SARS-CoV-2 Antibody Levels

**Figure 1:**
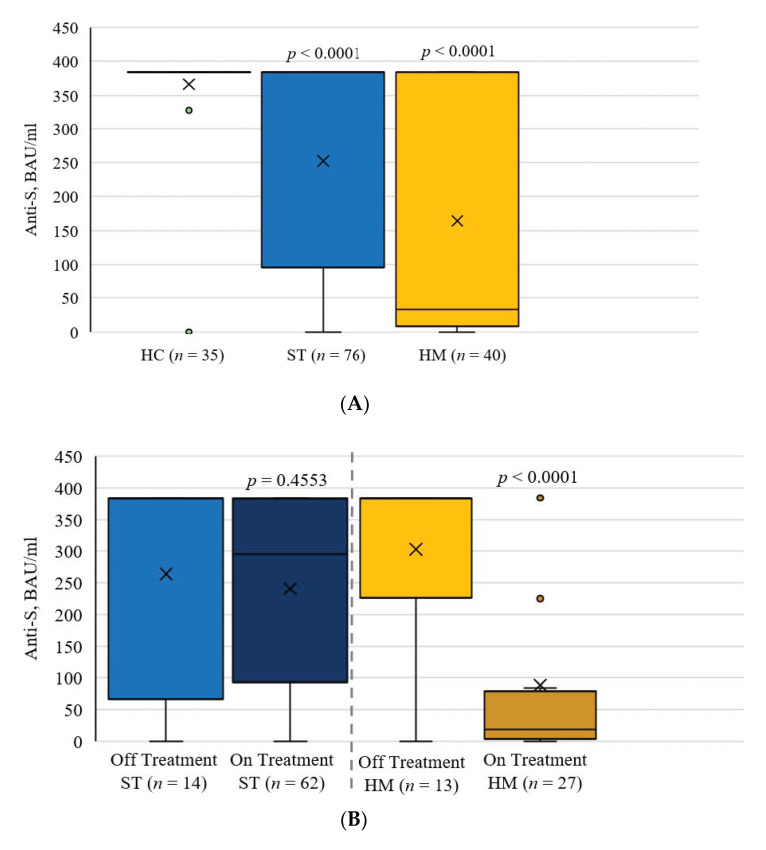

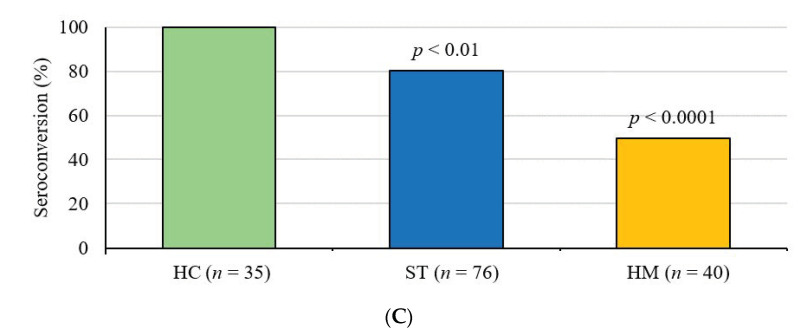
(**A**) Anti-spike Antibody Concentrations in Healthy Controls and Cancer Patients Following Two Doses of SARS-CoV-2 Vaccine. (**B**) Anti-spike Antibody Levels in Cancer Patients On and Off Treatment. (**C**) Frequency of Seroconversion Rate in Healthy Patients and Cancer Patients According to 35.2 BAU/mL cutoff. *p*-values as determined by (**A**) Tukey test against healthy controls, (**B**) Tukey test against off treatment group, and (**C**) Chi-square test against healthy controls. HC: healthy controls; ST: solid tumor; HM: hematological malignancy.

Following two doses of a SARS-CoV-2 vaccine, the mean serum anti-spike antibody levels were 382.4 BAU/mL (±9.4) in the control group, 265.8 BAU/mL (±145.7) in patients with solid cancer, and 168.2 BAU/mL (±172.9) in patients with hematological malignancies (Figure 1A). Observed differences were significant when comparing the control group to both cancer groups (*p* ≤ 0.0001). When comparing anti-spike antibody levels between cancer patients on treatment to those off treatment, the levels were significantly different for hematological malignancies (*p* < 0.0001) but not for solid cancers (*p* = 0.4553, Figure 1B). The Frequency of Seroconversion Rate in Healthy Patients and Cancer Patients was compared in Figure 1C using a cutoff of 35.2 BAU/mL (recommended by the ELISA manufacturer). Patients with solid and hematological malignancies demonstrated poor seroconversion rates, at 84.2% (*p* < 0.01) and 53% (*p* < 0.001), respectively (Figure 1C).

### 3.5. SARS-CoV-2 Antibody Level Comparison with Closely Matched Dosing Interval

**Figure 2:**
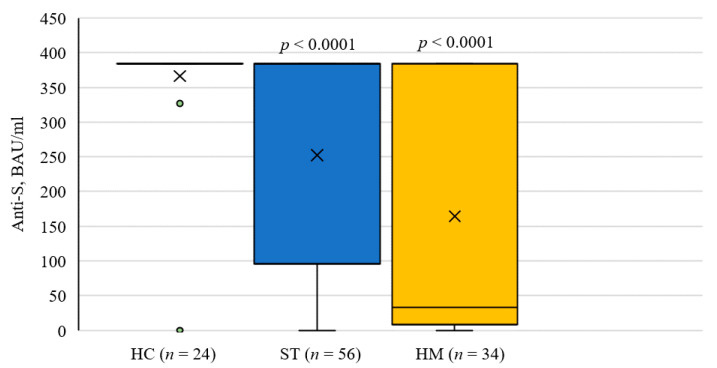
Anti-spike Antibody Concentrations in Healthy Controls and Cancer Patients with Closely Matched Dosing Intervals (Average 60 Days versus 54 Days, Respectively), Following Two Doses of SARS-CoV-2 Vaccine. *p*-values are determined by Tukey test against healthy controls. HC: healthy controls; ST: solid tumor; HM: hematological malignancy.

As there is evidence that longer dosing intervals have been associated with higher titer antibody responses [11], further analysis was performed with closely matched dosing intervals between cancer patients and healthy controls (average interval 54 days in cancer patient versus 60 days in the control, *p* > 0.05). Results were essentially unchanged, showing identical patterns (Figure 2).

## 4. Discussion

The COVID-19 pandemic has caused significant mortality and morbidity amongst immunocompromised patients. Limiting exposure and increasing vaccinations have been seen as two of the pillars of control in this setting.

In Ontario, as in much of Canada, changes in vaccine recommendations are constantly occurring. Cancer patients were amongst priority groups identified not only for vaccination, but also for shorter dosing intervals than non-cancer patients, which is reflected in the dosing intervals in this study. In addition, cancer patients may be prioritized for a third dose, as is currently recommended in Ontario for patients receiving systemic therapy. Understanding seroconversion rates and antibody protection in cancer patients in the Canadian context with the heterogeneity of dosing intervals, vaccines, cancers, and treatments is vitally important in managing the risk for these patients going forward in the COVID-19 pandemic.

We observed that anti-spike antibody concentrations were significantly lower in cancer patients than in healthy controls, and that patients with hematological malignancies receiving systemic therapy demonstrated a particularly impaired serological response. For patients receiving anti-CD20 based therapy, almost all patients had no discernible response at all. This is in accordance with recent literature [5,6,7]. In hematologic patients who were on active treatment but not receiving anti-CD20 treatment, a significant proportion also had an impaired serologic response. This group includes a broad range of diseases and treatments, including Hodgkin Lymphoma patients on chemotherapy such as ABVD, myeloma patients on treatments such as carfilzomib/dexamethasone, MDS patients on azacytidine, and chronic leukemia/lymphoma patients on agents such as ibrutinib, dasatinib, and hydroxyurea. Although this represents a very heterogenous group of hematologic malignancies and treatment, grouping them together was done, as guidance on third doses and other preventative strategies will pragmatically require grouping.

For patients with solid tumours, particularly those receiving cytotoxic therapy or cytotoxic therapy plus other therapies, a substantial amount did not seroconvert. However, for those with non-cytotoxic therapy, rates of seroconversion were similar to those on no therapy, including immunotherapy based, targeted therapy based, and hormonal therapy based.

In Ontario, Public Health Ontario has prioritized third dose vaccination for patients with hematologic malignancies, and, for patients with solid tumours, those receiving systemic therapy (excluding hormonal therapy). In addition, both the U.S. Food and Drug Administration and Health Canada have authorized the use of Pfizer-BioNTech and Moderna vaccines as booster doses in those 18 years of age and older at least 6 months after completion of the primary series. Our ELISA results are consistent with a potential lower level of protection after two doses in these patients. Given the extremely poor levels of seroconversion after two doses in certain groups—i.e., anti-CD20 treated patients, and hematologic patients on treatment—it is unclear if even a third dose will be effective in providing immunity. While a third dose is reasonable, it may still be essential for these groups to continue non-vaccine based preventative measures until a time such as third dose efficacy is established.

The antibody levels in our study differed from previous studies. The median concentration of antibody levels in our healthy control is 382.4 BAU/mL, whereas it was reported as 0.2 SU/mL [12] and 7160 AU/mL [5]. This may reflect different serological assays used, which renders results incomparable. Standardized and comparable serological testing is essential to evaluate humoral immunity post vaccination. We suggest that all methods should be traceable to the WHO International Standard for anti-SARS-CoV-2 immunoglobulin (NIBSC code 20/136), as is our method [13].

There is evidence that for some vaccines, longer dosing intervals have been associated with higher titer antibody responses [11]. Even though robust humoral responses were observed in the controls, cancer patients with closely matched dosing intervals were not able to generate adequate humoral responses. Therefore, we believe the better humoral immune response in the healthy control group is not due to longer dosing intervals.

The level of serological response post COVID-19 immunization that could render protection is unknown. From other immunization programs, this protection level ranges from 4 to 64 times of the seroconversion concentration [4]. Therefore, in our analysis (shown in Table 2), we arbitrarily chose 4 and 8 times of seroconversion concentration to explore the impact of various cutoffs on potential protection. This analysis demonstrated how various cutoffs could categorize patients as with or without immunity differently. For example, while there was no statistical difference in the antibody concentration between solid cancer patients without systemic therapy and healthy controls, the possible protection rates in such cancer patients were lower than the healthy control, especially if a higher cutoff was used. Statistical analysis was not performed as there was no scientific evidence yet to support either 4× or 8× as the cutoff for immunity. A large prospective epidemiological study is required to determine the serological threshold for possible immunity against further infection. We still want to caution the use of seroconversion in the study of immunity as it is unlikely to be a strong correlation for protection. Future research utilizing seroconversion as a correlate for immunity should be cautious in the interpretation of these values.

## 5. Limitations

The heterogeneity of vaccine types and schedules, and the sample size, did not allow for the comparison of the efficacy of various vaccines. Polymerase chain reaction specific for SARS-CoV-2 was not performed to rule out asymptomatic infection in the participants. The focus on humoral immunity may not reflect long term immunity in the form of memory B cells or in the T-cell response. This may be of particular relevance in patients undergoing effector B cell depleting therapy such as rituximab. Studies to assess T-cell immunity using assays are underway.

## 6. Conclusions

After two doses of the SARS-CoV-2 vaccination, patients with solid and hematological malignancies demonstrated impaired serological responses. This was particularly prominent if there was cytotoxic chemotherapy or systemic therapy in solid and hematological cancer, respectively. The level of serological response post COVID-19 immunization that could render protection is unknown. Therefore, certain cancer patients with no chemotherapy or systemic therapy may still be at risk for infection, especially if higher concentration of antibody levels are required for immunity.

## Figures and Tables

**Table 1:** Baseline Characteristics of Study Participants.

Characteristic	No. (%)	
	Patients (*n* = 116)	Controls (*n* = 35)
Age, median (range)	68 (24–91)	60 (43–79)
Sex		
Male	41 (35.3)	12 (34.3)
Female	75 (64.7)	23 (65.7)
Solid Tumours	76 (65.5)	N/A
Breast	26 (22.4)	
Gastrointestinal	21 (18.1)	
Lung	12 (10.3)	
Melanoma	7 (6.0)	
Genitourinary	5 (4.3)	
Gynecologic	3 (2.6)	
Other	2 (1.7)	
Hematologic Malignancy	40 (34.5)	
Lymphoma	21 (18.1)	
Leukemia	6 (5.2)	
Multiple myeloma	5 (4.4)	
Other	8 (6.9)	
Cancer treatment within 3 months of participation	89 (76.7)	N/A
Vaccine Received		
Pfizer-NBiotech (two doses)	84 (72.4)	12 (34.3)
AstraZeneca (two doses)	11 (9.5)	4 (11.4)
Moderna (two doses)	10 (8.6)	1 (2.9)
Mixed doses	11 (9.5)	18 (51.4)
Days between 1st and 2nd dose, mean (SD)	50 (±24)	66 (±14) *
Days between 2nd dose and blood collection, mean (SD)	44 (±27)	53 (±17)

* Further analysis discussed in 3.5.

**Table 2:** Anti-spike Antibody Concentration and Seroconversion Rates in Solid Tumour and Hematologic Patients following Two Doses of COVID-19 Vaccine, According to Treatment Type.

Participant Group Treatment Type	Patients (*n*)	Anti-S Concentration, BAU/mL		Seroconversion Rate per Cutoff, *n* (*%*)			*p*-Value (If < 0.05)
		Mean (±SD)	Median	35.2 BAU/mL	140.8 BAU/mL	281.6 BAU/mL	
Healthy Controls	35	382.4 (9.4)	384	35 (100)	35 (100)	35 (100)	
Solid Tumour	76	265.8 (145.7) *	384	64 (84)	56 (74)	45 (59)	* < 0.0001 * < 0.01
No systemic therapy	14	315 (156)	381	14 (100)	12 (86)	10 (64)	
Cytotoxic chemotherapy +	30	195 (156) */#	154	23 (77)	17 (57)	15 (50)	
Immunotherapy alone	14	300 (138)	384	14 (100)	11 (79)	10 (72)	
Targeted therapy alone	12	331 (126)	381	11 (92)	10 (83)	10 (83)	
Hormonal therapy alone	6	358 (64)	384	6 (100)	6 (100)	5 (83)	
Hematologic	40 13 11 16	168.2 (172.9) * 340 (116) 41 (113) */** 117 (144) */**	72 384 4 48	21 (53) 12 (93) 1 (9) 8 (50)	17 (43) 12 (93) 1 (9) 4 (25)	15 (38) 11 (86) 1 (9) 3 (19)	* < 0.0001 */** < 0.01 */** < 0.01
No active therapy							
Anti-CD20 therapy							
Systemic therapy(non-anti-CD20)							

* indicates comparison to healthy controls, using one-way ANOVA test with post-hoc Tukey test. ** indicates comparison to no therapy group within the same cancer type, using one-way ANOVA test with post-hoc Tukey test. # *p* = 0.056 if directly comparing cytotoxic chemotherapy to no therapy, and *p* = 0.24 when using Tukey test that accounts for multiple comparisons. No statistical differences between each treatment to no systemic therapy in the solid cancer group. + Cytotoxic chemotherapy alone or cytotoxic chemotherapy plus immunotherapy.

## Data Availability

The data presented in this study are available on request from the corresponding author.
